# A prospective study of hepatic safety of statins used in very elderly patients

**DOI:** 10.1186/s12877-019-1361-2

**Published:** 2019-12-16

**Authors:** Meizi Guo, Junli Zhao, Yingjiu Zhai, Panpan Zang, Qing Lv, Dongya Shang

**Affiliations:** 1 Department of Gereology, Shanghai University of Medicine & Health Sciences Affiliated Zhoupu Hospital, Zhou Yuan Road 1500, Pudong New Area, Shanghai, 201318 China; 2 Department of Nephrology, Shanghai University of Medicine & Health Sciences Affiliated Zhoupu Hospital, Shanghai, 201318 China

**Keywords:** Statins, Hepatotoxicity, Very elderly

## Abstract

**Background:**

Statins play an important role in the care of patients with cardiovascular disease and have a good safety record in clinical practice. Hepatotoxicity is a barrier that limits the ability of primary care physicians to prescribe statins for patients with elevated liver transaminase values and/or underlying liver disease. However, limited population-based data are available on the use of statin therapy and on the hepatotoxicity of statins in very elderly patients. This prospective study evaluated the liver enzyme elevation during statin therapy in very elderly patients (≥80 years old).

**Methods:**

Patients with hypercholesterolemia (LDL-C levels ≥3.4 and < 5.7 mmol/L), atherosclerosis, coronary heart disease (CHD), or a CHD-risk equivalent were enrolled and received once-daily statin treatment. Multivariate logistic regression models were used to study the impact of age, gender, hepatitis B infection, fatty liver disease, biliary calculus, other chronic diseases, drug kinds, alcohol abuse, statin variety, and statin dose variables.

**Results:**

A total of 515 consecutive patients ranging from 80 to 98 years old were included in the analysis. These patients were treated with simvastatin, fluvastatin, pravastatin, rosuvastatin, or atorvastatin. Twenty-four patients (4.7, 95% CI 2.7–6.6) showed an increase in their hepatic aminotransferase levels. No significant difference of hepatic aminotransferase elevation rates was observed in different statin treatment groups. The incidence of mild, moderate, and severe elevation of aminotransferase levels was 62.5% (15/24), 29.2% (7/24), and 8.3% (2/24), respectively. None of the patients developed hepatic failure. Nine patients with moderate or severe aminotransferase elevations discontinued therapy. The time of onset of hepatic aminotransferase elevation ranged from 2 weeks to 6 months after statin treatment. The onset of hepatic aminotransferase elevation was within 1 month for 70.8% of patients. The patients took 2 weeks to 3 months to recover their liver function after statin therapy cessation. Multivariate analysis identified chronic hepatitis B infection and alcohol consumption as independent factors associated with the hepatic response to statins: OR, 12.83; 95% CI (4.36–37.759) and OR, 2.736; 95% CI (1.373–5.454), respectively.

**Conclusion:**

The prevalence of elevated transaminases was higher than published data in very elderly patients. Overall, statin treatment is safe for patients ≥80 years old.

## Background

It is well known that a high blood level of the low-density lipoprotein-cholesterol (LDL-C) is a major risk factor that contributes to cardiovascular diseases (CVD). Many clinical trials have shown that patients with CVD can benefit from using statins to lower LDL-C levels [1–5]. Lipid-lowering therapy, specifically statins, has become a cornerstone of treatment for dyslipidemia due to their effects on LDL-C levels. As such, statin therapy is endorsed by various guidelines as the first-line pharmacotherapeutic approach for reducing LDL-C levels and CVD risk when they are not adequately controlled by lifestyle changes such as better diet, weight control, and exercise. As physicians, we often encounter concerns about the potential side effects of cholesterol treatment, including myopathy, rhabdomyolysis, and hepatic injury. The risk of hepatic injury caused by statins is reported to be about 1–3%, similar to that of patients taking a placebo [6–9]. Despite extensive data documenting the safety of statins, primary care physicians harbor significant hepatotoxicity concerns, and these concerns act as a barrier to the utilization of statins especially for elderly patients aged 80 years or older. The aim of this prospective study is to evaluate the liver enzyme elevation during statin therapy in these elderly patients.

## Methods

### Trial design

This was a 12-month, prospective study conducted in the Shanghai University of Medicine & Health Sciences Affiliated Zhoupu Hospital (Shanghai, China) from January 2014 to December 2015. The study was approved by the Medical Ethics Committees of Shanghai University of Medicine & Health Sciences Affiliated Zhoupu Hospital (2014-C-053-E01). All patients signed informed consent documents. Patients were treated with simvastatin, fluvastatin, pravastatin, rosuvastatin or atorvastatin by physician’s choice. To initiate treatment, physicians prescribed the standard dose of statins (40 mg of simvastatin, 40 mg of fluvastatin, 40 mg of pravastatin, 10 mg of rosuvastatin, or 20 mg of atorvastatin). If LDL-C levels were < 1.8 mmol/L (70 mg/dL) for 3 months, the statin dose was reduced by half.

### Inclusion and exclusion criteria

Patients who met all of the following criteria were recruited. All patients were more than 80 years old, and suffered from hypercholesterolemia, atherosclerosis, history of coronary heart disease (CHD) or a CHD risk equivalent. CHD risk equivalents include clinical manifestations of noncoronary forms of atherosclerotic disease (peripheral arterial disease, abdominal aortic aneurysm, and carotid artery disease, ie, transient ischemic attacks, stroke of carotid origin, or > 50% obstruction of a carotid artery), diabetes, and ≥ 2 risk factors with 10-year risk > 20% for hard CHD [10, 11]. The mean level of 2 most recent fasting LDL-C was between 3.4 mmol/L (130 mg/dL) and 5.7 mmol/L (220 mg/dL).

Patients who met one or more of the following criteria were not enrolled. The exclusion criteria were (a) history of statin-induced myopathy or a serious hypersensitivity to statins, (b) history of malignancy, (c) severe congestive heart failure (New York Heart Association class IIIb or IV), (d) current active liver disease (liver function is abnormal), (e) unexplained creatine kinase (CK) ≥ 3 × ULN, (f) serum creatinine > 176 μmol/L (2.0 mg/dL).

### Objectives

This prospective study was designed to evaluate the hepatic safety of statins and find risk factors for hepatotoxicity of statins in the oldest-old population (≥80 years old). Demographic and clinical data were collected for each patient. Potential risk factors for statin treatment included age, gender, hepatitis B infection, fatty liver disease, biliary calculus, other chronic diseases (diabetes, hypertension, obesity, et. al), commonly used grug kinds (hypoglycemic drugs, antihypertensives, aspirin, β-adrenoceptor blockers, et. al), alcohol use (no drinking, mild-moderate drinking, or heavy drinking), statin variety (simvastatin, fluvastatin, pravastatin, rosuvastatin, and atorvastatin), and statin dose (low dose or standard dose).

Liver function, including alanine aminotransferase (ALT) and aspartate aminotransferase (AST) levels at baseline (before starting statin therapy), was assessed at 1 week, 2 weeks, 1 month, 2 months, 3 months, 6 months, 9 months and 12 months after the initiation of statin therapy. Patients with serum AST > 40 IU/L or ALT > 40 IU/L were considered to have elevated transaminases because the upper limit of normal (ULN) for AST and ALT for our laboratory was 40 IU/L. The elevations in liver biochemistries during the follow-up were defined and categorized depending on the patient’s baseline levels of serum aminotransferase. For this study’s purposes, “mild” elevations in liver biochemistries was defined as elevations of AST and/or ALT less than 3 times ULN (< 3 × ULN, < 120 IU/L) in patients with normal baseline enzymes. “Moderate” elevations in liver biochemistries was defined as elevations of AST and/or ALT from 3 times up to 10 times ULN (3 × ULN-10 × ULN, 120-400 IU/L) in patients with normal baseline enzymes. “Severe” elevations in liver biochemistries was defined as the development of serum bilirubin > 3 mg/dL (regardless of transaminase levels) or elevations in AST and/or ALT greater than 10 times ULN (≥10 × ULN, ≥400 IU/L) in patients with normal baseline enzymes [6, 12]. During the study period, statins were available at the Zhoupu Hospital pharmacy.

### Sample size

The sample size for confidence intervals for a single proportion was calculated using SAS software. A sample size between 334 and 568 produces a two-sided 95% confidence interval with a width between 0.04 and 0.03 when the sample proportion is 0.03. A total of 568 patients who met all of the criteria were recruited. We excluded participants who had poor physical function (*n* = 53) in the period of observation.

### Statistical analyses

Data validity procedures, database management, and statistical analyses were performed using SAS software. Basic descriptive statistics, including means, standard deviations (SDs), ranges, and percentages, were used to characterize the study patients. We used binary logistic regression analysis to find risk factors first. Odds ratios (ORs) and 95% confidence intervals (CIs) were derived from the univariate analysis and multivariate logistic regression models. Comparisons with a *P* value < 0.05 were considered statistically significant.

## Results

### Study subjects

Baseline clinical demographics are provided in Table 1 and Additional file 1. A total of 515 consecutive patients were included in the pooled analysis, with ages ranging from 80 to 98 years old. The mean age was 83.8 ± 3.4 (SD) years. There were 410 men and 105 women included in the study (ratio 3.9:1), with average ages of 84.0 and 83.2 years, respectively. The following numbers of patients were treated with: Simvastatin 20–40 mg/d, *n* = 98[19.0%]; fluvastatin 40 mg/d, *n* = 116[22.5%]; pravastatin 10–20 mg/d, *n* = 80[15.5%]; rosuvastatin 5–10 mg/d, *n* = 85[16.5%]; atorvastatin 10–20 mg/d, *n* = 136[26.0%].

**Table 1:** Demographic and clinical characteristics of the study population (*n =* 515)

Variables	Percent (*N*)
Age (years)	83.8 ± 3.4
80 ≃ 85	63.5% (327)
85 ≃ 90	29.5% (152)
≥ 90	7% (36)
Sex	
Male	79.6% (410)
Female	20.4% (105)
Hepatitis B	
Yes	4.3% (22)
No	95.7% (493)
Fatty liver	
Yes	9.1% (47)
No	90.9% (468)
Biliary calculus	
Yes	17.1% (88)
No	82.9% (427)
Other diseases^a^	
≤ 2	6% (31)
3 ≃ 5	41.2% (212)
≥ 5	52.8% (272)
Other drugs^b^	
< 5	19.2% (99)
5 ≃ 10	71.5% (368)
≥ 10	9.3% (48)
Drinking habits^c^	
No drinking	95.3% (491)
Mild to moderate	2.7% (14)
Heavy	1.9% (10)
Statin variety	
Simvastatin	19.1% (98)
Fluvastatin	22.5% (116)
Pravastatin	15.5% (80)
Rosuvastatin	16.5% (85)
Atorvastatin	26.4% (136)
Statin dose	
Low	13.6% (70)
Standard	86.4% (445)

^a^Including hypertension, diabetes, cerebral infarction, atrial fibrillation, chronic bronchitis, chronic obstructive pulmonary disease, chronic gastritis, osteoporosis, benign prostatic hyperplasia, and biliary calculus

^b^Including antihypertensive agents, antidiabetic agents, clopidogrel, aspirin, beta-blockers, isosorbide mononitrate, trimetazidine, citicoline, cilostazol, warfarin, digoxin, ambroxol, aminophylline, rebamipide, rabeprazole, calcitriol, calcium carbonate, oral bisphosphonates, finasteride, tamsulosin, and digestive enzymes

^c^No drinking: not drinking in a previous year; light drinkers: current use of 3 drinks per week; moderate drinkers: current use of 3 to 7 drinks per week for women, and 3 to 14 drinks per week for men; heavy drinkers: current use of more than 7 drinks per week for women and 14 drinks per week for men

### Liver enzyme elevation

Twenty-four patients experienced increases in their hepatic aminotransferase levels, and the total rate of persistent elevation in hepatic aminotransferase levels was 4.7% (Tables 2 and 3). For each individual satin, the total rate was: 6.1% (6/98) for simvastatin, 6.9% (8/116) for fluvastatin, 5.0% (4/80) for pravastatin, 1.2% (1/85) for rosuvastatin, and 3.7% (5/136) for atorvastatin (Table 2). The incidence of mild, moderate, and severe aminotransferase elevation was 62.5% (15/24), 29.2% (7/24), and 8.3% (2/24), respectively (Table 3). None of the patients developed hepatic failure. Nine patients with moderate or severe aminotransferase elevations discontinued therapy. The time of onset of hepatic aminotransferase elevation ranged from 2 weeks to 6 months following the initiation of statin treatment (Table 3). The onset of hepatic aminotransferase elevation occurred within 1 month for 70.8% (17/24) of patients (Table 3). Patients required 2 weeks to 3 months to recover from their liver function after cessation of statin therapy (Table 3).

**Table 2:** Associations between liver enzyme elevation and various demographic and clinical variables

Characteristics	Presence of liver enzyme elevation		*P* value	Odds ratio (95% CI)
	Yes	No		
	(*n* = 24, 4.7%)	(*n* = 491, 95.3%)		
Age (years)	82.6 ± 3.8^a^	83.9 ± 3.4^a^		
80 ≃ 85	19(3.7%)^b^	308(59.8%)		1.000
85 ≃ 90	3(0.6%)	149(28.9%)	0.075	0.326(0.095, 1.120)
≥ 90	2(0.4%)	34(6.6%)	0.950	0.954(0.213, 4.271)
Sex				
Male	20(3.9%)	390(75.7%)		1.000
Female	4(0.8%)	101(19.6%)	0.644	0.772(0.258, 2.310)
Hepatitis B				
No	16(3.1%)	477(92.6%)		1.000
Yes	8(1.6%)	14(2.7%)	< 0.001	17.036(6.259, 46.371)
Fatty liver				
No	16(3.1%)	450(87.4%)		1.000
Yes	8(1.6%)	41(8.0%)	0.009	3.659(1.376, 9.726)
Biliary calculus				
No	16(3.1%)	411(79.8%)		1.000
Yes	8(1.6%)	80(15.5%)	0.036	2.570(1.064, 6.206)
Other diseases				
≤ 2	1(0.2%)	30(5.8%)		1.000
3 ≃ 5	9(1.7%)	203(39.4%)	0.790	1.330(0.163, 10.875)
≥ 5	14(2.7%)	258(50.1%)	0.644	1.628(0.207, 12.820)
Other drugs				
< 5	5(1.0%)	94(18.3%)		1.000
5 ≃ 10	16(3.1%)	352(68.3%)	0.765	0.855(0.305, 2.393)
≥ 10	3(0.6%)	45(8.7%)	0.764	1.253(0.287, 5.477)
Drinking habits				
No drinking	8(1.6%)	483(93.8%)		1.000
Mild to moderate	11(2.1%)	3(0.6%)	0.425	1.585(0.511, 4.914)
Heavy	5(1.0%)	5(1.0%)	< 0.001	27.733(7.245,106.164)
Statin variety				
Simvastatin	6(1.2%)	92(17.5%)		1.000
Fluvastatin	8(1.6%)	108(21.0%)	0.820	1.136(0.380, 3.393)
Pravastatin	4(0.8%)	76(14.8%)	0.747	0.807(0.220, 2.964)
Rosuvastatin	1(0.2%)	84(16.3%)	0.119	0.183(0.022, 1.548)
Atorvastatin	5(1.0%)	131(25.4%)	0.388	0.585(0.173, 1.975)
Statin dose				
Low	4(0.8%)	66(12.8%)		1.000
Standard	20(3.9%)	425(82.5%)	0.653	0.776(0.257, 2.342)

^a^data are expressed as mean ± SD. ^b^all percentages in the table are of the total samples (*n* = 515)

**Table 3:** Twenty-four patients with hepatic aminotransferase elevation after statin treatment

No.	Age	Sex	Statin	Before treatment		After treatment		Elevation levels	Liver enzyme elevation time	Recovery time
				ALT	AST	ALT	AST			
1	80 ≃ 85	M	Simvastatin	18	20	69	39	Mild	2 weeks	/
2	80 ≃ 85	M	Fluvastatin	21	19	70	37	Mild	1 month	/
3	80 ≃ 85	M	Simvastatin	16	16	165	143	Moderate	1 month	3 months
4	80 ≃ 85	F	Fluvastatin	14	18	49	51	Mild	1 month	/
5	80 ≃ 85	M	Atorvastatin	22	16	123	121	Moderate	3 months	1 month
6	80 ≃ 85	M	Fluvastatin	13	17	98	83	Mild	2 weeks	/
7	85 ≃ 90	M	Rosuvastatin	18	19	87	40	Mild	2 weeks	/
8	80 ≃ 85	M	Simvastatin	12	15	182	244	Moderate	1 month	1 month
9	≥90	M	Simvastatin	13	18	34	42	Mild	2 weeks	/
10	80 ≃ 85	M	Atorvastatin	27	22	1017	511	Severe	3 months	1 month
11	80 ≃ 85	M	Fluvastatin	10	11	66	71	Mild	2 weeks	/
12	80 ≃ 85	M	Simvastatin	15	16	261	301	Moderate	2 months	2 weeks
13	85 ≃ 90	F	Fluvastatin	23	25	51	49	Mild	3 months	/
14	80 ≃ 85	F	Pravastatin	19	17	107	48	Mild	2 months	/
15	80 ≃ 85	M	Fluvastatin	21	14	125	133	Moderate	2 weeks	1 month
16	80 ≃ 85	F	Simvastatin	34	24	979	696	Severe	6 months	1 month
17	≥90	M	Fluvastatin	15	20	47	54	Mild	1 month	/
18	85 ≃ 90	M	Pravastatin	16	13	70	43	Mild	1 month	/
19	80 ≃ 85	M	Pravastatin	13	14	57	51	Mild	1 month	/
20	80 ≃ 85	M	Atorvastatin	16	15	75	48	Mild	2 months	/
21	80 ≃ 85	M	Atorvastatin	10	13	44	53	Mild	2 weeks	/
22	80 ≃ 85	M	Atorvastatin	24	24	76	80	Mild	1 month	/
23	80 ≃ 85	M	Fluvastatin	26	25	267	222	Moderate	1 month	2 weeks
24	80 ≃ 85	M	Pravastatin	31	23	300	260	Moderate	1 month	1 month

*M* male, *F* female, *ALT* alanine aminotransferase, *AST* aspartate aminotransferase

### Risk factors of liver enzyme elevation

The variables age, gender, hepatitis B infection, fatty liver disease, biliary calculus, other chronic diseases, drug kinds, alcohol abuse, statin variety, and statin dose were included in the multivariate logistic regression analysis. No hepatitis C infection was observed in this study. Multivariate analysis identified chronic hepatitis B virus infection and alcohol use as independent risk factors associated with the liver enzyme elevation: OR, 12.83; 95% CI (4.36 to 37.759); *P* < 0.001; and OR, 2.736; 95% CI (1.373 to 5.454); *P* < 0.01, respectively (Table 4).

**Table 4:** Risk factors of liver enzyme elevation during statin therapy by multivariate analysis

Factor	Wald χ2	*P* value	Odds ratio	95% CI for OR	
				Lower	Upper
Hepatitis B	21.469	.000	12.830	4.360	37.759
Alcohol abuse	8.177	.004	2.736	1.373	5.454

## Discussion

Cardiovascular disease (CVD) was the leading cause of death, with a higher prevalence in older adults. Greater than 80% of CVD-related mortality occurs in patients ≥65 years old. There are many reports suggesting that lowering LDL-C reduces the risk of CVD. Statins are the first-line therapies for dyslipidemia and specify target LDL-C levels, but they are all hepatically cleared and can cause elevations in liver biochemistries. The potential risk for hepatotoxicity limited the use of statins [13–17]. This is especially true for patients over 80 years old. Thus, there is a treatment paradox in the elderly: the more the risk of vascular disease, the less frequently statin therapy is initiated. To ascertain the role of age in the risk of hepatotoxicity during statin therapy, our study selected very elderly patients, whose ages ranged from 80 to 98 years (mean age of 83.8 years). We found that there is no correlation between age and liver enzyme elevation during statin therapy in patients older than 80 years old. This is supported by multiple studies in different countries in other age range [16, 18, 19].

Elderly people often suffer from more than one chronic disease such as diabetes, heart failure, and cognitive impairment. The co-occurrence of two but often more than two medical conditions within a single person is called multimorbidity. Drug-induced liver injury is a major cause of liver injury. Drug metabolism, distribution and elimination change with age [20]. The elderly experience increased drug distribution and decreased hepatic clearance, which makes them more sensitive to polypharmacy which can lead to drug-induced liver injury. Hepatocellular injury and cholestasis are two of the most severe manifestations of drug-induced liver injury, accounting for nearly half of all hepatotoxic disease [21, 22]. It has been shown that elderly people who used concomitant drugs were more likely to experience cholestatic-type drug-induced liver injury [23]. In our study, simple factor analysis showed that biliary tract diseases are related to drug-induced liver injury; however, multivariate logistic regression analysis showed that these two diseases are unrelated (Tables 2 and 4).

Polypharmacy is common among frail, elderly patients (more than 50% of those 75 to 84 years old use 5–9 drugs per day) [24]. Age-dependent changes that occur during one’s lifespan can reduce liver mass, hepatic blood flow, protein synthesis, influence pharmacokinetics, and result in altered sensitivity to drugs. Multivariate analysis in our study showed no correlation between polypharmacy and statin-induced liver injury. It is important to note that all patients in the current study were not taking fibrates, a class of cholesterol-lowering drugs (Table 1).

Statins are the most well-known lipid-lowering medication in the arena of CVD. An increase in the level of liver enzymes is one of the most important adverse side effects of statin use, and can lead to liver damage [25]. A meta-analysis showed that patients had a higher likelihood of transaminase elevation when they were treated with high doses of atorvasatin, fluvastatin, lovastatin, and simvastatin [26]. Our results showed that statin-induced liver injury was not associated with any specific type of statin (Table 1). Through extensive clinical experience we have found that statins are most effective for treating CVD patients when a standard dose is used. In our study, we used the standard dose of statins instead of a higher dose. When blood lipid levels decreased below our national guideline or were in the normal range before treatment, we used a low dose of statins to treat the patients. Our results show that there was no relationship between statin dose and statin-induced liver injury.

Some concerns have been raised regarding the widespread use of statin therapy. One of common challenges is multimorbidity, diabetes mellitus, obesity, and dyslipidemia, which are the main features of nonalcoholic fatty liver disease (NAFLD). Cardiovascular events may also coexist with other chronic liver diseases, such as fatty liver disease (FLD), Hepatitis B and C, and liver cirrhosis. The clearance of all statins occurs in the liver and can cause elevations of some liver biochemistries [23], thus there is a concern about the increased risk of liver injury in patients taking statins. There are some studies that indicate that when patients with hyperlipidaemia and other liver diseases, like NAFLD, are treated with statins, elevations of ALT and liver enzyme levels occur in serum [17, 27]. Therefore, the safety of statin treatment in patients with NAFLD has aroused concern. However, based on previous case reports, treatment with statins is safe and may actually improve liver function [28, 29]. In our results, multivariate analysis showed that FLD patients undergoing statin treatment do not appear to have an increased risk of hepatic damage (OR, 3.659; 95% CI (1.376 to 9.726); *P* = 0.009; Table 2). However, hepatitis patients taking statins did experience hepatic damage (OR, 17.036; 95% CI (6.259 to 46.371); *P* < 0.001), even though they had normal liver function before statin treatment (Tables 2 and 3).

Moderate consumption of alcohol increases high density lipoprotein (HDL-C) levels. When patients use statins with 30 g alcohol/d, the level HDL-C was significantly increased compared to the control group [30]. Excessive amounts of alcohol worsen liver function, which can increase the impact of statins on the liver. Our results indicate that heavy drinkers taking statins are more susceptible to liver injury (Table 2).

### Limitations

The current study was subject to some limitations. During the observation period, there was no supply of pitavastatin in our hospital, so there was a lack of data on liver damage caused by the use of pitavastatin. In addition, the causal relationship between liver enzyme elevations and Hepatitis B infection or alcohol abuse during statin therapy should be confirmed by more studies as liver enzyme elevations were common in patients with Hepatitis B infection and alcohol abuse. Finally, this study did not include a control group. Satins were widely used in the very elderly people in China. We set a non-statin control group at the beginning of the study (*N* = 188). However, 37.2% participants (*N* = 70) in the control group were excluded due to the use of statins in the period of the observation, leading to the result that the risk factors, such as age, gender, kinds of diseases and drugs in the control group didn’t match the experimental group.

## Conclusion

To date, there has been not enough evidence to indicate that the incidence of hepatotoxicity or elevation of aminotransferase levels is higher in elderly patients receiving statins compared with younger patients. After a year of follow-up of the 515 patients in our study, the data suggest that the total rate of persistent aminotransferase elevation is 4.7% (95% CI 2.7–6.6). There is no correlation between drug-induced liver injury and age, gender, biliary calculus, fatty liver, comorbidity, polypharmacy, or the statin variety. It is safe to use the standard dose of statins in patients aged 80 years or older. Recently, the FDA has recommended the revision of labeling instructions for statins and has suggested that routine monitoring of liver enzymes in patients taking statins is unnecessary. However, in our opinion, monitoring liver function is necessary if the patient has chronic liver disease or a history of alcohol abuse.

## Supplementary information

**Additional file 1: Table S1.** Clinical characteristics of total population.

## Data Availability

The datasets used for the current study are available from the corresponding author upon reasonable request.

## Abbreviations

ALT: Alanine aminotransferase
AST: Aspartate aminotransferase
CHD: Coronary heart disease
CVD: Cardiovascular diseases
FLD: Fatty liver disease
HDL-C: High-density lipoprotein-cholesterol
HMG-CoA: 3-hydroxy-3-methylglutaryl co-enzyme A
LDL-C: Low-density lipoprotein-cholesterol
NAFLD: Nonalcoholic fatty liver disease
ULN: Upper limit of normal
