# Supplementary material for: A prospective study of hepatic safety of statins used in very elderly patients
Source: BMC Geriatr. 2019 Dec 16;19:352. doi: 10.1186/s12877-019-1361-2 (PMC6915904; doi:10.1186/s12877-019-1361-2)
Supplement: Supplementary file 1 — Additional file 1: Table S1. Clinical characteristics of total population. [file 12877_2019_1361_MOESM1_ESM.pdf]

No.	Age	Sex	Hepatitis B	Fatty Liver	Biliary calculus	Compli-cations	Other drugs	Drinking habits	Statin variety	Statin dose	Hepatic damage
1	80≃85	Male	Yes	Yes	No	≥5	5≃10	No drinking	Atorvastatin	Standard	No
2	80≃85	Male	No	No	No	3≃5	5≃10	No drinking	Simvastatin	Standard	No
3	80≃85	Male	No	No	No	≥5	5≃10	No drinking	Simvastatin	Standard	No
4	80≃85	Female	No	No	No	3≃5	<5	No drinking	Fluvastatin	Standard	No
5	80≃85	Male	No	No	No	≥5	5≃10	No drinking	Fluvastatin	Standard	No
6	80≃85	Female	No	No	No	≥5	5≃10	No drinking	Fluvastatin	Standard	No
7	85≃90	Male	No	No	No	≥5	5≃10	No drinking	Simvastatin	Standard	No
8	80≃85	Male	No	No	No	3≃5	5≃10	No drinking	Atorvastatin	Standard	No
9	≥90	Male	No	No	No	3≃5	5≃10	No drinking	Atorvastatin	Standard	No
10	80≃85	Male	No	No	No	3≃5	5≃10	No drinking	Fluvastatin	Standard	No
11	80≃85	Male	No	No	No	≥5	5≃10	No drinking	Atorvastatin	Low	No
12	80≃85	Male	No	No	Yes	≥5	5≃10	No drinking	Simvastatin	Standard	No
13	85≃90	Male	No	No	No	≥5	5≃10	No drinking	Fluvastatin	Standard	No
14	80≃85	Male	No	No	No	3≃5	5≃10	No drinking	Simvastatin	Standard	No
15	80≃85	Male	No	No	No	≥5	5≃10	No drinking	Rosuvastatin	Standard	No
16	85≃90	Male	No	No	Yes	≥5	5≃10	No drinking	Atorvastatin	Standard	No
17	80≃85	Male	No	No	No	≥5	5≃10	No drinking	Simvastatin	Standard	No
18	80≃85	Male	No	No	No	3≃5	5≃10	No drinking	Fluvastatin	Standard	No
19	85≃90	Male	No	No	No	≥5	5≃10	No drinking	Simvastatin	Standard	No
20	80≃85	Male	No	No	No	3≃5	<5	No drinking	Atorvastatin	Standard	No
21	85≃90	Male	No	No	No	≥5	5≃10	No drinking	Simvastatin	Standard	No
22	85≃90	Male	No	No	No	≥5	5≃10	No drinking	Atorvastatin	Standard	No
23	85≃90	Female	No	Yes	No	≥5	5≃10	No drinking	Fluvastatin	Standard	No
24	≥90	Male	No	No	No	3≃5	<5	No drinking	Pravastatin	Standard	No
25	80≃85	Male	No	No	No	3≃5	5≃10	No drinking	Fluvastatin	Standard	No
26	80≃85	Male	Yes	No	Yes	≥5	5≃10	No drinking	Simvastatin	Standard	No
27	85≃90	Male	No	No	Yes	≥5	5≃10	No drinking	Atorvastatin	Low	No
28	80≃85	Male	No	No	No	3≃5	5≃10	No drinking	Rosuvastatin	Standard	No
29	80≃85	Female	No	No	No	≥5	5≃10	No drinking	Fluvastatin	Standard	No
30	85≃90	Female	No	No	No	≥5	<5	No drinking	Atorvastatin	Standard	No
31	85≃90	Male	No	No	No	≥5	5≃10	No drinking	Pravastatin	Standard	No
32	85≃90	Male	No	No	No	≥5	5≃10	No drinking	Simvastatin	Standard	No
33	85≃90	Female	No	No	Yes	3≃5	<5	No drinking	Simvastatin	Standard	No
34	85≃90	Male	No	No	No	≥5	5≃10	No drinking	Atorvastatin	Standard	No
35	80≃85	Male	No	No	No	≥5	5≃10	No drinking	Fluvastatin	Standard	No
36	85≃90	Male	No	No	No	≥5	5≃10	No drinking	Rosuvastatin	Standard	No
37	80≃85	Male	No	No	No	≥5	<5	No drinking	Pravastatin	Standard	No
38	80≃85	Male	No	No	No	≥5	<5	No drinking	Simvastatin	Standard	No
39	80≃85	Female	No	No	No	≥5	5≃10	No drinking	Atorvastatin	Standard	No
40	80≃85	Male	No	No	No	3≃5	<5	No drinking	Simvastatin	Standard	No
41	80≃85	Female	No	No	No	≥5	5≃10	No drinking	Atorvastatin	Standard	No
42	85≃90	Male	No	No	No	≥5	5≃10	No drinking	Fluvastatin	Standard	No
43	85≃90	Male	No	No	No	3≃5	5≃10	No drinking	Atorvastatin	Standard	No
44	80≃85	Male	No	No	No	≥5	5≃10	No drinking	Simvastatin	Standard	No
45	80≃85	Female	No	Yes	No	≥5	5≃10	No drinking	Atorvastatin	Low	No
46	85≃90	Male	No	No	No	≥5	5≃10	No drinking	Rosuvastatin	Standard	No
47	80≃85	Female	No	No	No	3≃5	<5	No drinking	Fluvastatin	Standard	No

No.	Age	Sex	Hepatitis B	Fatty Liver	Biliary calculus	Compli-cations	Other drugs	Drinking habits	Statin variety	Statin dose	Hepatic damage
48	85≃90	Male	No	No	No	≥5	<5	No drinking	Simvastatin	Standard	No
49	80≃85	Male	No	No	No	≥5	5≃10	No drinking	Fluvastatin	Standard	No
50	80≃85	Male	No	No	No	≥5	<5	No drinking	Atorvastatin	Standard	No
51	≥90	Male	No	No	No	3≃5	<5	No drinking	Fluvastatin	Standard	No
52	85≃90	Male	No	No	No	3≃5	5≃10	No drinking	Fluvastatin	Standard	No
53	85≃90	Male	No	No	No	3≃5	5≃10	No drinking	Simvastatin	Standard	No
54	80≃85	Female	No	No	Yes	≥5	5≃10	No drinking	Atorvastatin	Standard	No
55	80≃85	Male	No	No	No	≥5	5≃10	No drinking	Simvastatin	Standard	No
56	80≃85	Male	No	No	Yes	3≃5	5≃10	No drinking	Simvastatin	Standard	No
57	85≃90	Male	No	No	No	≤2	<5	No drinking	Atorvastatin	Standard	No
58	80≃85	Male	No	No	No	3≃5	5≃10	No drinking	Simvastatin	Low	No
59	80≃85	Female	No	No	No	≥5	5≃10	No drinking	Pravastatin	Standard	No
60	80≃85	Male	No	No	Yes	≥5	<5	No drinking	Atorvastatin	Standard	No
61	80≃85	Male	No	No	No	≥5	5≃10	No drinking	Simvastatin	Standard	No
62	80≃85	Female	No	No	No	3≃5	≥10	No drinking	Simvastatin	Standard	No
63	85≃90	Male	No	No	No	≥5	<5	No drinking	Atorvastatin	Standard	No
64	85≃90	Female	No	No	No	3≃5	5≃10	No drinking	Rosuvastatin	Standard	No
65	80≃85	Female	No	No	No	3≃5	5≃10	No drinking	Fluvastatin	Standard	No
66	80≃85	Male	No	No	No	≥5	5≃10	No drinking	Simvastatin	Standard	No
67	85≃90	Male	No	No	Yes	≥5	5≃10	No drinking	Atorvastatin	Standard	No
68	80≃85	Male	No	No	Yes	≥5	5≃10	No drinking	Simvastatin	Standard	No
69	≥90	Male	No	No	Yes	≥5	<5	No drinking	Pravastatin	Standard	No
70	80≃85	Female	No	No	No	3≃5	5≃10	No drinking	Atorvastatin	Standard	No
71	85≃90	Male	No	No	No	≥5	5≃10	No drinking	Pravastatin	Low	No
72	≥90	Male	No	No	No	3≃5	5≃10	No drinking	Fluvastatin	Standard	No
73	80≃85	Male	No	No	No	≥5	5≃10	No drinking	Simvastatin	Standard	No
74	80≃85	Male	No	No	No	≥5	5≃10	No drinking	Fluvastatin	Standard	No
75	85≃90	Male	No	No	No	≥5	5≃10	No drinking	Simvastatin	Standard	No
76	80≃85	Male	No	No	No	3≃5	<5	No drinking	Atorvastatin	Standard	No
77	80≃85	Male	No	No	No	3≃5	5≃10	No drinking	Fluvastatin	Standard	No
78	80≃85	Male	No	No	No	3≃5	5≃10	No drinking	Simvastatin	Standard	No
79	85≃90	Male	No	No	No	3≃5	<5	No drinking	Atorvastatin	Low	No
80	≥90	Female	No	No	No	3≃5	<5	No drinking	Rosuvastatin	Standard	No
81	80≃85	Male	No	No	No	≥5	<5	No drinking	Simvastatin	Standard	No
82	85≃90	Male	No	No	Yes	≥5	5≃10	No drinking	Simvastatin	Low	No
83	≥90	Female	No	No	No	≥5	5≃10	No drinking	Atorvastatin	Standard	No
84	85≃90	Female	No	No	No	≥5	5≃10	No drinking	Simvastatin	Standard	No
85	85≃90	Male	No	Yes	Yes	≥5	5≃10	No drinking	Fluvastatin	Standard	No
86	85≃90	Male	No	No	Yes	≥5	5≃10	No drinking	Atorvastatin	Standard	No
87	≥90	Male	No	No	No	≥5	5≃10	No drinking	Fluvastatin	Standard	No
88	85≃90	Male	No	No	No	≥5	5≃10	No drinking	Pravastatin	Standard	No
89	85≃90	Male	No	No	Yes	≥5	5≃10	No drinking	Atorvastatin	Standard	No
90	≥90	Male	No	No	No	≥5	5≃10	No drinking	Fluvastatin	Standard	No
91	80≃85	Female	No	No	No	3≃5	<5	No drinking	Atorvastatin	Low	No
92	80≃85	Male	No	No	No	≥5	5≃10	No drinking	Fluvastatin	Standard	No
93	80≃85	Female	No	No	No	≥5	5≃10	No drinking	Pravastatin	Standard	No
94	85≃90	Male	Yes	No	Yes	≥5	5≃10	No drinking	Atorvastatin	Standard	No

No.	Age	Sex	Hepatitis B	Fatty Liver	Biliary calculus	Compli-cations	Other drugs	Drinking habits	Statin variety	Statin dose	Hepatic damage
95	85≃90	Male	No	No	No	3≃5	5≃10	No drinking	Fluvastatin	Standard	No
96	80≃85	Male	No	No	No	≥5	<5	No drinking	Atorvastatin	Standard	No
97	80≃85	Male	No	No	Yes	3≃5	5≃10	No drinking	Fluvastatin	Standard	No
98	80≃85	Male	No	Yes	No	≥5	5≃10	No drinking	Simvastatin	Standard	No
99	80≃85	Male	No	No	No	≥5	5≃10	No drinking	Pravastatin	Standard	No
100	80≃85	Male	No	No	No	≥5	5≃10	No drinking	Atorvastatin	Low	No
101	85≃90	Male	No	No	No	≥5	<5	No drinking	Simvastatin	Standard	No
102	80≃85	Male	No	No	No	3≃5	<5	No drinking	Fluvastatin	Standard	No
103	80≃85	Female	No	No	No	≥5	5≃10	No drinking	Atorvastatin	Standard	No
104	80≃85	Female	No	No	No	3≃5	5≃10	No drinking	Fluvastatin	Standard	No
105	80≃85	Male	No	No	No	≥5	5≃10	No drinking	Fluvastatin	Standard	No
106	≥90	Female	No	No	Yes	≥5	5≃10	No drinking	Atorvastatin	Standard	No
107	80≃85	Male	No	No	No	≥5	5≃10	No drinking	Rosuvastatin	Standard	No
108	80≃85	Male	No	No	Yes	≥5	5≃10	No drinking	Simvastatin	Low	No
109	85≃90	Male	No	No	Yes	≥5	5≃10	No drinking	Atorvastatin	Standard	No
110	85≃90	Male	No	No	No	≥5	<5	No drinking	Rosuvastatin	Standard	No
111	85≃90	Male	No	No	No	3≃5	<5	No drinking	Fluvastatin	Standard	No
112	85≃90	Male	No	No	No	3≃5	<5	No drinking	Rosuvastatin	Standard	No
113	85≃90	Male	No	No	No	≥5	<5	No drinking	Rosuvastatin	Low	No
114	85≃90	Male	No	No	No	≥5	5≃10	No drinking	Fluvastatin	Standard	No
115	85≃90	Male	No	No	No	≥5	5≃10	No drinking	Atorvastatin	Standard	No
116	85≃90	Male	No	No	No	3≃5	<5	No drinking	Simvastatin	Standard	No
117	80≃85	Female	No	No	Yes	≥5	5≃10	No drinking	Fluvastatin	Standard	No
118	80≃85	Female	No	No	No	≥5	5≃10	No drinking	Fluvastatin	Standard	No
119	80≃85	Male	No	No	Yes	3≃5	<5	No drinking	Rosuvastatin	Standard	No
120	80≃85	Male	No	No	Yes	3≃5	<5	No drinking	Simvastatin	Low	No
121	80≃85	Male	No	No	No	≥5	5≃10	No drinking	Atorvastatin	Standard	No
122	80≃85	Male	No	No	No	3≃5	5≃10	No drinking	Pravastatin	Standard	No
123	80≃85	Male	No	No	No	3≃5	<5	No drinking	Fluvastatin	Standard	No
124	85≃90	Male	No	No	No	3≃5	5≃10	No drinking	Atorvastatin	Standard	No
125	85≃90	Male	No	No	Yes	≥5	5≃10	No drinking	Simvastatin	Low	No
126	80≃85	Male	No	No	No	≥5	5≃10	No drinking	Fluvastatin	Standard	No
127	80≃85	Male	No	No	No	≥5	5≃10	No drinking	Fluvastatin	Standard	No
128	85≃90	Male	No	Yes	No	≥5	5≃10	No drinking	Atorvastatin	Low	No
129	80≃85	Male	No	No	Yes	3≃5	5≃10	No drinking	Fluvastatin	Standard	No
130	80≃85	Male	No	No	No	≥5	5≃10	No drinking	Atorvastatin	Standard	No
131	85≃90	Male	No	No	No	3≃5	<5	No drinking	Rosuvastatin	Standard	No
132	80≃85	Male	No	No	No	≥5	5≃10	No drinking	Fluvastatin	Standard	No
133	80≃85	Male	No	No	Yes	≥5	<5	No drinking	Simvastatin	Standard	No
134	85≃90	Male	No	No	No	≥5	5≃10	No drinking	Atorvastatin	Standard	No
135	80≃85	Male	No	No	No	3≃5	5≃10	No drinking	Atorvastatin	Standard	No
136	80≃85	Male	No	No	No	3≃5	5≃10	No drinking	Simvastatin	Standard	No
137	80≃85	Male	No	No	No	≥5	5≃10	No drinking	Pravastatin	Low	No
138	80≃85	Male	No	No	Yes	≥5	5≃10	No drinking	Atorvastatin	Standard	No
139	85≃90	Male	No	No	No	3≃5	<5	No drinking	Fluvastatin	Standard	No
140	85≃90	Male	No	No	Yes	3≃5	<5	No drinking	Fluvastatin	Standard	No
141	85≃90	Female	No	No	No	≥5	5≃10	No drinking	Atorvastatin	Standard	No

No.	Age	Sex	Hepatitis B	Fatty Liver	Biliary calculus	Compli-cations	Other drugs	Drinking habits	Statin variety	Statin dose	Hepatic damage
142	80≃85	Male	No	No	Yes	≥5	5≃10	No drinking	Simvastatin	Low	No
143	80≃85	Male	No	No	No	≤2	<5	No drinking	Fluvastatin	Standard	No
144	≥90	Male	No	No	No	≥5	5≃10	No drinking	Atorvastatin	Low	No
145	80≃85	Male	No	Yes	No	≥5	5≃10	No drinking	Rosuvastatin	Standard	No
146	≥90	Male	No	No	No	≥5	5≃10	No drinking	Pravastatin	Standard	No
147	80≃85	Male	No	No	No	≥5	<5	No drinking	Rosuvastatin	Standard	No
148	80≃85	Male	No	No	Yes	3≃5	<5	No drinking	Atorvastatin	Standard	No
149	80≃85	Female	No	No	No	≥5	<5	No drinking	Simvastatin	Standard	No
150	80≃85	Male	No	No	No	3≃5	5≃10	No drinking	Fluvastatin	Standard	No
151	85≃90	Male	No	No	No	3≃5	5≃10	No drinking	Pravastatin	Standard	No
152	80≃85	Male	No	No	No	3≃5	5≃10	No drinking	Simvastatin	Standard	No
153	80≃85	Male	No	No	No	3≃5	5≃10	No drinking	Atorvastatin	Standard	No
154	80≃85	Male	No	No	No	≥5	5≃10	No drinking	Fluvastatin	Standard	No
155	80≃85	Male	No	No	Yes	3≃5	5≃10	No drinking	Simvastatin	Standard	No
156	85≃90	Male	No	No	No	≥5	5≃10	No drinking	Atorvastatin	Low	No
157	85≃90	Male	No	No	No	≥5	5≃10	No drinking	Fluvastatin	Standard	No
158	80≃85	Male	No	No	Yes	≥5	5≃10	No drinking	Simvastatin	Standard	No
159	80≃85	Male	No	No	No	3≃5	5≃10	No drinking	Simvastatin	Low	No
160	80≃85	Male	No	No	No	3≃5	<5	No drinking	Rosuvastatin	Standard	No
161	80≃85	Male	No	No	No	≥5	<5	No drinking	Atorvastatin	Standard	No
162	80≃85	Male	No	No	No	3≃5	<5	No drinking	Fluvastatin	Standard	No
163	80≃85	Male	No	No	No	3≃5	5≃10	No drinking	Atorvastatin	Standard	No
164	85≃90	Male	No	No	No	3≃5	5≃10	No drinking	Pravastatin	Standard	No
165	80≃85	Male	No	No	No	≤2	<5	No drinking	Atorvastatin	Low	No
166	85≃90	Male	No	No	No	≥5	5≃10	No drinking	Simvastatin	Standard	No
167	85≃90	Male	No	No	No	≥5	5≃10	No drinking	Rosuvastatin	Standard	No
168	80≃85	Male	No	No	No	≥5	5≃10	No drinking	Atorvastatin	Standard	No
169	85≃90	Female	No	No	No	3≃5	<5	No drinking	Atorvastatin	Low	No
170	80≃85	Male	Yes	No	No	3≃5	<5	No drinking	Fluvastatin	Standard	No
171	80≃85	Male	No	No	No	3≃5	5≃10	No drinking	Simvastatin	Standard	No
172	80≃85	Male	No	No	Yes	≥5	5≃10	No drinking	Simvastatin	Low	No
173	80≃85	Male	No	No	No	≥5	5≃10	No drinking	Atorvastatin	Standard	No
174	80≃85	Male	No	No	No	≥5	5≃10	No drinking	Fluvastatin	Standard	No
175	80≃85	Male	No	No	No	3≃5	5≃10	No drinking	Simvastatin	Low	No
176	80≃85	Male	No	No	No	≥5	5≃10	No drinking	Fluvastatin	Standard	No
177	85≃90	Male	No	No	No	3≃5	<5	No drinking	Fluvastatin	Standard	No
178	85≃90	Male	No	No	No	≥5	<5	No drinking	Atorvastatin	Standard	No
179	80≃85	Female	No	No	No	≥5	5≃10	No drinking	Fluvastatin	Standard	No
180	80≃85	Female	No	No	No	≥5	5≃10	No drinking	Rosuvastatin	Standard	No
181	80≃85	Female	No	No	No	3≃5	5≃10	No drinking	Pravastatin	Low	No
182	85≃90	Male	No	No	No	3≃5	<5	No drinking	Pravastatin	Standard	No
183	85≃90	Male	No	No	No	≥5	5≃10	No drinking	Atorvastatin	Standard	No
184	80≃85	Male	No	No	No	≥5	5≃10	No drinking	Fluvastatin	Standard	No
185	80≃85	Male	No	No	Yes	≥5	5≃10	No drinking	Pravastatin	Standard	No
186	80≃85	Male	No	No	No	3≃5	5≃10	No drinking	Atorvastatin	Standard	No
187	≥90	Male	No	No	No	3≃5	5≃10	No drinking	Fluvastatin	Standard	No
188	80≃85	Male	No	No	No	≥5	5≃10	No drinking	Simvastatin	Standard	No

No.	Age	Sex	Hepatitis B	Fatty Liver	Biliary calculus	Compli-cations	Other drugs	Drinking habits	Statin variety	Statin dose	Hepatic damage
189	85≃90	Male	No	No	No	≥5	5≃10	No drinking	Rosuvastatin	Standard	No
190	≥90	Female	No	No	No	≥5	5≃10	No drinking	Atorvastatin	Standard	No
191	80≃85	Male	No	No	No	≥5	5≃10	No drinking	Fluvastatin	Standard	No
192	85≃90	Female	No	No	No	3≃5	<5	No drinking	Simvastatin	Standard	No
193	85≃90	Female	No	No	Yes	3≃5	<5	No drinking	Atorvastatin	Standard	No
194	80≃85	Female	No	No	No	3≃5	5≃10	No drinking	Rosuvastatin	Standard	No
195	80≃85	Male	No	No	No	≥5	5≃10	Mild to moderate	Atorvastatin	Standard	No
196	80≃85	Male	No	No	No	≥5	5≃10	No drinking	Fluvastatin	Standard	No
197	80≃85	Male	No	No	No	≥5	5≃10	No drinking	Pravastatin	Standard	No
198	85≃90	Male	No	No	No	3≃5	<5	No drinking	Atorvastatin	Standard	No
199	80≃85	Female	No	No	No	3≃5	<5	No drinking	Simvastatin	Standard	No
200	80≃85	Male	No	No	No	≥5	5≃10	No drinking	Atorvastatin	Standard	No
201	80≃85	Male	No	No	No	3≃5	5≃10	No drinking	Simvastatin	Standard	No
202	80≃85	Male	No	No	No	≥5	5≃10	No drinking	Rosuvastatin	Standard	No
203	80≃85	Male	No	Yes	No	3≃5	5≃10	No drinking	Atorvastatin	Standard	No
204	80≃85	Male	No	No	No	≥5	5≃10	No drinking	Simvastatin	Standard	No
205	80≃85	Male	No	No	No	3≃5	5≃10	No drinking	Fluvastatin	Standard	No
206	80≃85	Male	Yes	No	No	≥5	5≃10	No drinking	Fluvastatin	Standard	No
207	80≃85	Male	No	No	No	≤2	<5	No drinking	Atorvastatin	Standard	No
208	85≃90	Male	No	No	No	≥5	5≃10	No drinking	Fluvastatin	Standard	No
209	80≃85	Male	No	No	No	3≃5	5≃10	No drinking	Simvastatin	Standard	No
210	80≃85	Male	No	No	No	≤2	<5	No drinking	Simvastatin	Standard	No
211	85≃90	Male	No	No	No	3≃5	5≃10	No drinking	Pravastatin	Low	No
212	85≃90	Male	No	No	No	3≃5	5≃10	No drinking	Atorvastatin	Standard	No
213	85≃90	Male	No	No	No	3≃5	5≃10	No drinking	Pravastatin	Standard	No
214	85≃90	Male	No	No	No	3≃5	5≃10	No drinking	Simvastatin	Standard	No
215	80≃85	Male	No	No	No	3≃5	5≃10	No drinking	Fluvastatin	Standard	No
216	80≃85	Male	No	No	No	3≃5	5≃10	No drinking	Pravastatin	Standard	No
217	80≃85	Male	No	No	No	3≃5	<5	No drinking	Atorvastatin	Standard	No
218	85≃90	Male	No	No	No	3≃5	5≃10	No drinking	Simvastatin	Standard	No
219	≥90	Male	No	No	No	≥5	5≃10	No drinking	Fluvastatin	Standard	No
220	80≃85	Male	No	No	No	3≃5	<5	No drinking	Rosuvastatin	Standard	No
221	80≃85	Male	No	No	No	≤2	5≃10	No drinking	Atorvastatin	Standard	No
222	80≃85	Male	No	No	No	3≃5	5≃10	No drinking	Fluvastatin	Standard	No
223	80≃85	Male	No	No	No	3≃5	5≃10	No drinking	Simvastatin	Standard	No
224	80≃85	Male	No	No	No	≥5	5≃10	No drinking	Pravastatin	Standard	No
225	≥90	Male	No	No	Yes	≥5	<5	No drinking	Atorvastatin	Standard	No
226	80≃85	Male	No	No	Yes	3≃5	5≃10	No drinking	Fluvastatin	Standard	No
227	80≃85	Male	No	No	No	≥5	5≃10	No drinking	Rosuvastatin	Standard	No
228	85≃90	Female	No	No	No	≥5	≥10	No drinking	Atorvastatin	Standard	No
229	80≃85	Male	No	No	No	3≃5	5≃10	No drinking	Simvastatin	Standard	No
230	80≃85	Male	No	No	No	≥5	5≃10	No drinking	Fluvastatin	Standard	No
231	80≃85	Male	No	No	No	3≃5	5≃10	No drinking	Pravastatin	Standard	No
232	80≃85	Male	No	Yes	No	3≃5	5≃10	No drinking	Atorvastatin	Standard	No
233	80≃85	Male	No	No	No	≥5	5≃10	No drinking	Fluvastatin	Standard	No
234	80≃85	Male	No	No	No	3≃5	<5	No drinking	Rosuvastatin	Standard	No
235	80≃85	Male	No	No	No	≥5	5≃10	No drinking	Pravastatin	Standard	No

No.	Age	Sex	Hepatitis B	Fatty Liver	Biliary calculus	Compli-cations	Other drugs	Drinking habits	Statin variety	Statin dose	Hepatic damage
236	80≃85	Female	No	No	No	3≃5	5≃10	No drinking	Rosuvastatin	Standard	No
237	80≃85	Male	No	Yes	Yes	3≃5	5≃10	No drinking	Atorvastatin	Low	No
238	80≃85	Male	No	No	No	3≃5	5≃10	No drinking	Rosuvastatin	Standard	No
239	85≃90	Male	No	No	No	3≃5	5≃10	No drinking	Atorvastatin	Standard	No
240	80≃85	Male	No	No	No	3≃5	5≃10	No drinking	Fluvastatin	Standard	No
241	80≃85	Male	No	Yes	No	3≃5	<5	No drinking	Simvastatin	Standard	No
242	80≃85	Male	No	No	No	≥5	5≃10	No drinking	Simvastatin	Low	No
243	80≃85	Male	No	No	No	≤2	5≃10	No drinking	Atorvastatin	Standard	No
244	80≃85	Male	No	No	No	≤2	5≃10	No drinking	Simvastatin	Standard	No
245	80≃85	Male	No	No	No	3≃5	5≃10	No drinking	Atorvastatin	Standard	No
246	85≃90	Male	No	No	Yes	≥5	5≃10	No drinking	Fluvastatin	Standard	No
247	≥90	Male	No	Yes	Yes	≥5	5≃10	No drinking	Pravastatin	Standard	No
248	80≃85	Female	No	No	No	3≃5	5≃10	No drinking	Atorvastatin	Low	No
249	80≃85	Male	No	No	No	3≃5	5≃10	No drinking	Simvastatin	Low	No
250	80≃85	Male	No	No	No	3≃5	5≃10	No drinking	Atorvastatin	Standard	No
251	80≃85	Female	No	No	No	≥5	5≃10	No drinking	Fluvastatin	Standard	No
252	80≃85	Male	No	No	Yes	≥5	5≃10	No drinking	Pravastatin	Standard	No
253	80≃85	Female	No	No	No	3≃5	<5	No drinking	Fluvastatin	Standard	No
254	80≃85	Female	No	No	No	3≃5	5≃10	No drinking	Atorvastatin	Low	No
255	≥90	Male	No	No	No	3≃5	5≃10	No drinking	Fluvastatin	Standard	No
256	85≃90	Male	No	No	No	≥5	5≃10	No drinking	Atorvastatin	Low	No
257	85≃90	Male	No	No	No	≥5	5≃10	No drinking	Simvastatin	Standard	No
258	80≃85	Male	No	No	Yes	≥5	5≃10	No drinking	Fluvastatin	Standard	No
259	80≃85	Male	No	No	No	≤2	<5	No drinking	Rosuvastatin	Standard	No
260	≥90	Female	No	No	No	≥5	5≃10	No drinking	Atorvastatin	Low	No
261	85≃90	Male	No	No	Yes	≥5	5≃10	No drinking	Fluvastatin	Standard	No
262	80≃85	Female	No	No	No	≤2	5≃10	No drinking	Rosuvastatin	Standard	No
263	80≃85	Male	No	No	Yes	≥5	5≃10	No drinking	Atorvastatin	Standard	No
264	80≃85	Male	No	No	No	≥5	5≃10	No drinking	Fluvastatin	Standard	No
265	80≃85	Male	No	No	No	3≃5	<5	No drinking	Simvastatin	Standard	No
266	80≃85	Male	No	No	No	≥5	5≃10	No drinking	Fluvastatin	Standard	No
267	80≃85	Female	No	No	No	3≃5	5≃10	No drinking	Fluvastatin	Standard	No
268	80≃85	Male	No	No	No	≥5	5≃10	No drinking	Atorvastatin	Standard	No
269	80≃85	Male	No	No	No	≥5	5≃10	No drinking	Fluvastatin	Standard	No
270	80≃85	Male	No	No	Yes	3≃5	5≃10	No drinking	Fluvastatin	Standard	No
271	80≃85	Male	No	No	No	3≃5	5≃10	No drinking	Atorvastatin	Low	No
272	85≃90	Female	No	No	No	3≃5	<5	No drinking	Pravastatin	Standard	No
273	≥90	Male	No	Yes	No	≥5	5≃10	No drinking	Atorvastatin	Low	No
274	80≃85	Male	No	No	Yes	3≃5	5≃10	No drinking	Rosuvastatin	Low	No
275	80≃85	Male	No	No	No	3≃5	5≃10	No drinking	Rosuvastatin	Standard	No
276	80≃85	Male	No	No	No	≥5	5≃10	No drinking	Atorvastatin	Standard	No
277	80≃85	Female	No	Yes	No	≥5	5≃10	No drinking	Pravastatin	Standard	No
278	85≃90	Male	No	No	No	3≃5	5≃10	No drinking	Rosuvastatin	Standard	No
279	85≃90	Male	No	No	No	≤2	5≃10	No drinking	Atorvastatin	Standard	No
280	80≃85	Female	No	No	No	3≃5	5≃10	No drinking	Simvastatin	Standard	No
281	80≃85	Male	No	No	No	3≃5	<5	No drinking	Pravastatin	Standard	No
282	85≃90	Male	No	No	No	≤2	<5	No drinking	Atorvastatin	Low	No

No.	Age	Sex	Hepatitis B	Fatty Liver	Biliary calculus	Compli-cations	Other drugs	Drinking habits	Statin variety	Statin dose	Hepatic damage
283	85≃90	Male	No	No	No	≤2	<5	No drinking	Rosuvastatin	Standard	No
284	80≃85	Male	No	No	No	3≃5	5≃10	No drinking	Fluvastatin	Standard	No
285	80≃85	Male	No	Yes	No	3≃5	5≃10	No drinking	Pravastatin	Standard	No
286	85≃90	Male	No	No	No	3≃5	5≃10	No drinking	Atorvastatin	Low	No
287	85≃90	Male	No	No	No	≤2	5≃10	No drinking	Rosuvastatin	Standard	No
288	85≃90	Male	No	No	No	3≃5	5≃10	No drinking	Pravastatin	Standard	No
289	80≃85	Female	No	No	No	≥5	5≃10	No drinking	Atorvastatin	Standard	No
290	80≃85	Male	No	Yes	No	≥5	5≃10	No drinking	Rosuvastatin	Low	No
291	85≃90	Male	No	No	No	≥5	5≃10	No drinking	Atorvastatin	Standard	No
292	85≃90	Female	No	Yes	No	≥5	5≃10	No drinking	Simvastatin	Standard	No
293	≥90	Male	Yes	No	No	3≃5	5≃10	No drinking	Fluvastatin	Standard	No
294	85≃90	Female	No	No	No	≤2	<5	No drinking	Simvastatin	Standard	No
295	85≃90	Male	No	No	No	≥5	5≃10	No drinking	Atorvastatin	Low	No
296	80≃85	Male	No	No	No	3≃5	<5	No drinking	Fluvastatin	Standard	No
297	80≃85	Male	No	No	No	≥5	5≃10	No drinking	Rosuvastatin	Standard	No
298	80≃85	Male	No	Yes	No	≥5	5≃10	No drinking	Pravastatin	Standard	No
299	85≃90	Male	No	No	No	≥5	5≃10	No drinking	Atorvastatin	Low	No
300	80≃85	Female	No	No	No	≥5	5≃10	No drinking	Fluvastatin	Standard	No
301	80≃85	Male	No	No	No	3≃5	5≃10	No drinking	Fluvastatin	Standard	No
302	≥90	Male	No	No	No	3≃5	<5	No drinking	Fluvastatin	Standard	No
303	80≃85	Male	No	No	No	3≃5	<5	No drinking	Pravastatin	Standard	No
304	80≃85	Male	No	No	No	3≃5	<5	No drinking	Rosuvastatin	Standard	No
305	85≃90	Male	No	No	Yes	≥5	5≃10	No drinking	Fluvastatin	Standard	No
306	85≃90	Male	No	No	Yes	≥5	5≃10	No drinking	Pravastatin	Standard	No
307	80≃85	Male	No	No	No	≥5	5≃10	No drinking	Atorvastatin	Standard	No
308	80≃85	Female	No	No	No	≥5	≥10	No drinking	Fluvastatin	Standard	No
309	80≃85	Female	No	No	No	≥5	≥10	No drinking	Rosuvastatin	Low	No
310	80≃85	Male	No	No	No	3≃5	5≃10	No drinking	Atorvastatin	Low	No
311	85≃90	Male	No	No	No	3≃5	5≃10	No drinking	Simvastatin	Standard	No
312	80≃85	Female	No	No	No	≤2	5≃10	No drinking	Fluvastatin	Standard	No
313	≥90	Male	No	No	No	≥5	<5	No drinking	Rosuvastatin	Standard	No
314	80≃85	Female	No	No	Yes	≥5	5≃10	No drinking	Atorvastatin	Standard	No
315	85≃90	Male	No	No	No	3≃5	<5	No drinking	Fluvastatin	Standard	No
316	80≃85	Male	No	No	No	≥5	5≃10	No drinking	Pravastatin	Standard	No
317	80≃85	Male	No	No	No	3≃5	5≃10	No drinking	Atorvastatin	Standard	No
318	80≃85	Male	No	No	No	3≃5	5≃10	No drinking	Simvastatin	Low	No
319	80≃85	Male	No	No	Yes	≥5	5≃10	No drinking	Fluvastatin	Standard	No
320	85≃90	Male	No	No	No	≥5	<5	No drinking	Atorvastatin	Standard	No
321	80≃85	Male	No	No	No	≥5	5≃10	No drinking	Fluvastatin	Standard	No
322	80≃85	Male	No	Yes	No	3≃5	5≃10	No drinking	Fluvastatin	Standard	No
323	85≃90	Male	No	No	No	≥5	5≃10	No drinking	Atorvastatin	Low	No
324	85≃90	Male	No	No	No	≤2	5≃10	No drinking	Fluvastatin	Standard	No
325	80≃85	Male	No	No	Yes	≥5	5≃10	No drinking	Pravastatin	Standard	No
326	85≃90	Male	No	No	No	3≃5	<5	No drinking	Rosuvastatin	Low	No
327	80≃85	Male	No	No	No	3≃5	5≃10	No drinking	Atorvastatin	Standard	No
328	80≃85	Male	No	No	No	≥5	5≃10	No drinking	Pravastatin	Standard	No
329	80≃85	Male	No	Yes	Yes	≥5	5≃10	No drinking	Atorvastatin	Standard	No

No.	Age	Sex	Hepatitis B	Fatty Liver	Biliary calculus	Compli-cations	Other drugs	Drinking habits	Statin variety	Statin dose	Hepatic damage
330	80≃85	Male	No	No	No	≥5	<5	No drinking	Simvastatin	Low	No
331	80≃85	Female	No	No	No	≥5	5≃10	No drinking	Fluvastatin	Standard	No
332	80≃85	Male	No	No	Yes	≥5	5≃10	No drinking	Simvastatin	Standard	No
333	80≃85	Male	No	No	Yes	≥5	5≃10	No drinking	Rosuvastatin	Standard	No
334	80≃85	Male	No	No	No	≥5	5≃10	No drinking	Atorvastatin	Standard	No
335	80≃85	Male	No	No	No	≥5	5≃10	No drinking	Fluvastatin	Standard	No
336	85≃90	Male	No	No	No	≥5	5≃10	No drinking	Fluvastatin	Standard	No
337	80≃85	Female	No	No	No	3≃5	<5	No drinking	Pravastatin	Standard	No
338	≥90	Male	No	No	No	3≃5	<5	No drinking	Fluvastatin	Standard	No
339	80≃85	Male	No	Yes	Yes	≥5	5≃10	No drinking	Fluvastatin	Standard	No
340	80≃85	Male	No	No	No	≥5	5≃10	No drinking	Pravastatin	Standard	No
341	80≃85	Male	No	No	Yes	3≃5	5≃10	No drinking	Atorvastatin	Standard	No
342	80≃85	Male	No	No	Yes	≥5	5≃10	No drinking	Fluvastatin	Standard	No
343	85≃90	Male	No	No	Yes	≥5	5≃10	No drinking	Pravastatin	Standard	No
344	80≃85	Male	No	No	No	≤2	<5	No drinking	Pravastatin	Standard	No
345	80≃85	Male	No	Yes	Yes	≥5	5≃10	No drinking	Rosuvastatin	Low	No
346	80≃85	Female	No	No	No	≥5	5≃10	No drinking	Fluvastatin	Standard	No
347	85≃90	Female	No	No	No	≥5	5≃10	No drinking	Atorvastatin	Standard	No
348	80≃85	Male	No	No	No	≥5	<5	No drinking	Fluvastatin	Standard	No
349	80≃85	Male	No	No	No	3≃5	5≃10	No drinking	Atorvastatin	Standard	No
350	≥90	Male	No	Yes	No	≥5	5≃10	No drinking	Simvastatin	Standard	No
351	80≃85	Male	No	No	No	3≃5	5≃10	No drinking	Atorvastatin	Standard	No
352	80≃85	Female	No	No	No	3≃5	5≃10	No drinking	Simvastatin	Standard	No
353	80≃85	Male	Yes	No	Yes	≥5	5≃10	No drinking	Fluvastatin	Standard	No
354	80≃85	Male	No	No	No	≥5	5≃10	No drinking	Pravastatin	Standard	No
355	80≃85	Female	No	No	No	3≃5	5≃10	No drinking	Rosuvastatin	Standard	No
356	80≃85	Female	No	No	Yes	3≃5	5≃10	No drinking	Fluvastatin	Standard	No
357	80≃85	Female	No	No	Yes	3≃5	5≃10	No drinking	Atorvastatin	Standard	No
358	≥90	Male	No	No	No	≥5	5≃10	No drinking	Fluvastatin	Standard	No
359	85≃90	Male	No	No	No	3≃5	5≃10	No drinking	Pravastatin	Standard	No
360	≥90	Male	No	No	No	3≃5	5≃10	No drinking	Fluvastatin	Standard	No
361	80≃85	Male	No	No	No	≥5	5≃10	No drinking	Atorvastatin	Standard	No
362	80≃85	Male	No	No	No	3≃5	<5	No drinking	Fluvastatin	Standard	No
363	85≃90	Male	No	No	No	≥5	5≃10	No drinking	Fluvastatin	Standard	No
364	85≃90	Male	No	No	No	≥5	5≃10	No drinking	Simvastatin	Standard	No
365	≥90	Male	No	No	No	≥5	5≃10	No drinking	Fluvastatin	Standard	No
366	80≃85	Male	No	No	No	≥5	5≃10	No drinking	Fluvastatin	Standard	No
367	80≃85	Male	No	Yes	No	≥5	5≃10	No drinking	Fluvastatin	Standard	No
368	80≃85	Male	No	Yes	No	≥5	5≃10	No drinking	Pravastatin	Low	No
369	85≃90	Male	No	Yes	No	3≃5	5≃10	No drinking	Rosuvastatin	Standard	No
370	80≃85	Male	No	No	No	3≃5	<5	No drinking	Atorvastatin	Low	No
371	85≃90	Male	No	No	No	≥5	5≃10	No drinking	Simvastatin	Standard	No
372	85≃90	Male	No	No	No	≥5	≥10	No drinking	Simvastatin	Standard	No
373	85≃90	Male	No	No	No	≥5	≥10	No drinking	Fluvastatin	Standard	No
374	80≃85	Male	No	No	Yes	≥5	≥10	No drinking	Simvastatin	Standard	No
375	85≃90	Male	No	No	No	≥5	≥10	No drinking	Atorvastatin	Low	No
376	85≃90	Male	No	No	No	≤2	5≃10	No drinking	Simvastatin	Standard	No

No.	Age	Sex	Hepatitis B	Fatty Liver	Biliary calculus	Compli-cations	Other drugs	Drinking habits	Statin variety	Statin dose	Hepatic damage
377	80≃85	Male	No	No	No	≥5	≥10	No drinking	Atorvastatin	Standard	No
378	80≃85	Male	No	No	No	≥5	≥10	No drinking	Simvastatin	Standard	No
379	80≃85	Male	No	No	No	≥5	≥10	No drinking	Atorvastatin	Standard	No
380	80≃85	Male	No	No	No	≥5	≥10	No drinking	Rosuvastatin	Standard	No
381	85≃90	Male	No	No	No	3≃5	≥10	No drinking	Atorvastatin	Standard	No
382	80≃85	Female	No	No	No	3≃5	5≃10	No drinking	Simvastatin	Standard	No
383	80≃85	Male	No	No	No	3≃5	5≃10	No drinking	Atorvastatin	Standard	No
384	80≃85	Female	No	No	No	≤2	5≃10	No drinking	Rosuvastatin	Low	No
385	80≃85	Male	No	No	No	3≃5	5≃10	No drinking	Atorvastatin	Standard	No
386	80≃85	Female	No	No	No	3≃5	5≃10	No drinking	Rosuvastatin	Standard	No
387	85≃90	Female	No	No	No	≤2	5≃10	No drinking	Simvastatin	Standard	No
388	85≃90	Male	No	No	No	3≃5	5≃10	No drinking	Pravastatin	Standard	No
389	80≃85	Female	No	No	No	≥5	5≃10	No drinking	Atorvastatin	Standard	No
390	80≃85	Male	No	No	No	≥5	≥10	No drinking	Fluvastatin	Standard	No
391	85≃90	Female	No	No	No	≥5	5≃10	No drinking	Simvastatin	Standard	No
392	85≃90	Male	No	No	No	3≃5	5≃10	No drinking	Atorvastatin	Standard	No
393	≥90	Male	No	No	No	≥5	≥10	No drinking	Simvastatin	Standard	No
394	80≃85	Male	No	No	No	≥5	5≃10	No drinking	Simvastatin	Low	No
395	80≃85	Male	No	No	No	3≃5	≥10	No drinking	Rosuvastatin	Standard	No
396	85≃90	Male	No	No	Yes	≥5	≥10	No drinking	Atorvastatin	Standard	No
397	85≃90	Male	No	No	No	3≃5	5≃10	No drinking	Rosuvastatin	Standard	No
398	80≃85	Male	No	No	No	≥5	5≃10	No drinking	Atorvastatin	Standard	No
399	85≃90	Male	No	No	No	3≃5	5≃10	No drinking	Simvastatin	Standard	No
400	80≃85	Male	No	Yes	No	3≃5	5≃10	No drinking	Atorvastatin	Standard	No
401	≥90	Male	No	No	No	3≃5	5≃10	No drinking	Rosuvastatin	Standard	No
402	80≃85	Male	No	No	No	≥5	≥10	No drinking	Pravastatin	Standard	No
403	85≃90	Male	No	No	No	3≃5	≥10	No drinking	Pravastatin	Standard	No
404	80≃85	Female	No	No	No	3≃5	≥10	No drinking	Atorvastatin	Standard	No
405	85≃90	Female	No	No	No	3≃5	5≃10	No drinking	Simvastatin	Standard	No
406	80≃85	Female	No	No	No	3≃5	5≃10	No drinking	Atorvastatin	Standard	No
407	80≃85	Female	No	No	No	3≃5	5≃10	No drinking	Simvastatin	Standard	No
408	80≃85	Female	No	No	No	3≃5	5≃10	No drinking	Rosuvastatin	Standard	No
409	80≃85	Male	No	Yes	No	≤2	5≃10	No drinking	Pravastatin	Standard	No
410	80≃85	Male	No	No	No	3≃5	5≃10	No drinking	Atorvastatin	Standard	No
411	85≃90	Male	No	Yes	No	3≃5	<5	Heavy drinking	Rosuvastatin	Standard	No
412	80≃85	Female	No	No	No	≤2	5≃10	No drinking	Pravastatin	Standard	No
413	80≃85	Female	No	No	Yes	3≃5	5≃10	No drinking	Pravastatin	Standard	No
414	80≃85	Female	No	No	No	≤2	<5	No drinking	Rosuvastatin	Low	No
415	80≃85	Male	No	No	Yes	3≃5	<5	Mild to moderate	Rosuvastatin	Standard	No
416	80≃85	Male	No	No	No	3≃5	5≃10	No drinking	Pravastatin	Standard	No
417	80≃85	Male	No	No	No	≤2	5≃10	No drinking	Rosuvastatin	Low	No
418	85≃90	Male	No	No	No	≤2	5≃10	No drinking	Pravastatin	Standard	No
419	85≃90	Male	No	No	No	3≃5	5≃10	No drinking	Pravastatin	Standard	No
420	80≃85	Male	No	No	No	≤2	5≃10	No drinking	Rosuvastatin	Standard	No
421	85≃90	Male	No	Yes	Yes	≤2	5≃10	No drinking	Rosuvastatin	Low	No
422	80≃85	Male	No	Yes	No	3≃5	≥10	No drinking	Pravastatin	Standard	No
423	85≃90	Male	No	No	Yes	≥5	≥10	No drinking	Atorvastatin	Standard	No

No.	Age	Sex	Hepatitis B	Fatty Liver	Biliary calculus	Compli-cations	Other drugs	Drinking habits	Statin variety	Statin dose	Hepatic damage
424	80≃85	Female	No	No	No	≥5	≥10	No drinking	Rosuvastatin	Standard	No
425	85≃90	Male	No	No	No	≥5	≥10	No drinking	Pravastatin	Standard	No
426	80≃85	Male	No	Yes	Yes	≥5	≥10	No drinking	Atorvastatin	Standard	No
427	80≃85	Male	No	No	No	≥5	5≃10	No drinking	Simvastatin	Standard	No
428	85≃90	Female	No	No	No	3≃5	5≃10	No drinking	Rosuvastatin	Standard	No
429	85≃90	Male	No	No	No	3≃5	5≃10	No drinking	Atorvastatin	Standard	No
430	85≃90	Female	No	No	No	≥5	5≃10	No drinking	Pravastatin	Low	No
431	80≃85	Male	No	Yes	No	3≃5	<5	No drinking	Rosuvastatin	Standard	No
432	80≃85	Male	No	No	Yes	≥5	5≃10	No drinking	Atorvastatin	Standard	No
433	85≃90	Female	No	No	No	3≃5	5≃10	No drinking	Rosuvastatin	Standard	No
434	80≃85	Female	No	No	No	≥5	≥10	No drinking	Atorvastatin	Standard	No
435	85≃90	Male	No	No	No	≥5	≥10	No drinking	Rosuvastatin	Standard	No
436	80≃85	Male	No	Yes	Yes	≥5	≥10	No drinking	Atorvastatin	Standard	No
437	80≃85	Female	No	Yes	No	3≃5	<5	No drinking	Pravastatin	Standard	No
438	80≃85	Female	No	No	No	3≃5	<5	No drinking	Rosuvastatin	Low	No
439	80≃85	Female	No	No	No	3≃5	5≃10	No drinking	Pravastatin	Low	No
440	80≃85	Female	No	No	No	3≃5	5≃10	No drinking	Rosuvastatin	Standard	No
441	85≃90	Female	No	No	No	3≃5	5≃10	No drinking	Atorvastatin	Standard	No
442	80≃85	Male	No	No	No	≤2	5≃10	No drinking	Pravastatin	Standard	No
443	80≃85	Female	No	No	No	3≃5	5≃10	No drinking	Pravastatin	Standard	No
444	80≃85	Female	Yes	No	No	≥5	5≃10	No drinking	Rosuvastatin	Standard	No
445	85≃90	Male	Yes	Yes	No	≥5	5≃10	No drinking	Atorvastatin	Standard	No
446	85≃90	Male	Yes	Yes	No	≥5	5≃10	No drinking	Fluvastatin	Standard	No
447	85≃90	Male	No	Yes	No	≥5	≥10	No drinking	Rosuvastatin	Low	No
448	80≃85	Male	No	No	Yes	3≃5	≥10	No drinking	Pravastatin	Standard	No
449	≥90	Male	No	No	No	3≃5	5≃10	No drinking	Pravastatin	Standard	No
450	80≃85	Male	No	No	No	≥5	5≃10	No drinking	Rosuvastatin	Standard	No
451	80≃85	Male	No	No	No	3≃5	≥10	No drinking	Pravastatin	Standard	No
452	85≃90	Male	No	No	No	3≃5	5≃10	No drinking	Rosuvastatin	Low	No
453	80≃85	Male	No	No	No	≥5	≥10	No drinking	Pravastatin	Standard	No
454	85≃90	Male	No	No	No	3≃5	≥10	No drinking	Rosuvastatin	Low	No
455	80≃85	Female	No	No	No	≥5	≥10	No drinking	Pravastatin	Low	No
456	80≃85	Female	No	No	No	≥5	≥10	No drinking	Rosuvastatin	Standard	No
457	85≃90	Male	No	No	No	≥5	≥10	No drinking	Pravastatin	Standard	No
458	80≃85	Male	No	No	No	≥5	≥10	No drinking	Rosuvastatin	Standard	No
459	85≃90	Male	No	No	No	≥5	≥10	No drinking	Atorvastatin	Standard	No
460	80≃85	Male	No	No	No	3≃5	≥10	Heavy drinking	Rosuvastatin	Standard	No
461	85≃90	Male	No	No	No	3≃5	5≃10	No drinking	Pravastatin	Standard	No
462	≥90	Male	No	No	No	≥5	≥10	No drinking	Rosuvastatin	Standard	No
463	80≃85	Male	No	No	No	≥5	5≃10	No drinking	Pravastatin	Standard	No
464	85≃90	Male	No	No	No	≥5	5≃10	No drinking	Atorvastatin	Standard	No
465	85≃90	Female	No	No	No	≥5	5≃10	No drinking	Rosuvastatin	Standard	No
466	85≃90	Male	No	No	No	3≃5	5≃10	No drinking	Rosuvastatin	Standard	No
467	80≃85	Male	No	No	No	3≃5	5≃10	No drinking	Rosuvastatin	Standard	No
468	≥90	Male	No	No	No	≥5	5≃10	No drinking	Pravastatin	Low	No
469	80≃85	Female	No	No	Yes	≥5	5≃10	No drinking	Pravastatin	Standard	No
470	85≃90	Male	No	No	No	≤2	<5	No drinking	Atorvastatin	Standard	No

No.	Age	Sex	Hepatitis B	Fatty Liver	Biliary calculus	Compli-cations	Other drugs	Drinking habits	Statin variety	Statin dose	Hepatic damage
471	80≃85	Male	No	No	No	3≃5	5≃10	No drinking	Rosuvastatin	Standard	No
472	80≃85	Female	No	No	No	3≃5	≥10	No drinking	Atorvastatin	Standard	No
473	80≃85	Female	No	No	No	3≃5	5≃10	No drinking	Rosuvastatin	Standard	No
474	85≃90	Male	No	No	Yes	≥5	≥10	No drinking	Rosuvastatin	Standard	No
475	80≃85	Male	No	No	No	≥5	≥10	No drinking	Rosuvastatin	Standard	No
476	80≃85	Male	Yes	Yes	No	≥5	<5	Heavy drinking	Simvastatin	Standard	No
477	80≃85	Male	Yes	Yes	Yes	≥5	5≃10	Heavy drinking	Pravastatin	Standard	No
478	80≃85	Male	Yes	No	No	3≃5	<5	No drinking	Simvastatin	Standard	No
479	80≃85	Female	Yes	Yes	No	≥5	5≃10	No drinking	Simvastatin	Standard	No
480	80≃85	Male	No	No	No	3≃5	≥10	Mild to moderate	Rosuvastatin	Standard	No
481	80≃85	Male	No	No	No	3≃5	5≃10	Heavy drinking	Pravastatin	Standard	No
482	85≃90	Male	No	No	No	≥5	5≃10	No drinking	Pravastatin	Low	No
483	80≃85	Male	No	No	No	3≃5	5≃10	No drinking	Rosuvastatin	Standard	No
484	≥90	Male	No	No	No	3≃5	<5	No drinking	Pravastatin	Standard	No
485	80≃85	Male	No	No	Yes	≥5	<5	No drinking	Rosuvastatin	Standard	No
486	80≃85	Male	No	No	Yes	3≃5	5≃10	No drinking	Rosuvastatin	Standard	No
487	80≃85	Male	No	No	No	3≃5	5≃10	No drinking	Simvastatin	Standard	No
488	85≃90	Female	No	No	Yes	≥5	5≃10	No drinking	Pravastatin	Standard	No
489	80≃85	Female	No	No	No	≥5	5≃10	No drinking	Rosuvastatin	Low	No
490	80≃85	Male	No	No	Yes	≥5	5≃10	No drinking	Rosuvastatin	Standard	No
491	80≃85	Female	No	No	Yes	≥5	≥10	No drinking	Pravastatin	Low	No
492	80≃85	Male	Yes	Yes	Yes	≥5	5≃10	Heavy drinking	Simvastatin	Standard	Yes
493	80≃85	Male	Yes	No	Yes	≤2	<5	Mild to moderate	Fluvastatin	Standard	Yes
494	80≃85	Male	No	No	No	3≃5	5≃10	Mild to moderate	Simvastatin	Low	Yes
495	80≃85	Female	Yes	Yes	No	≥5	5≃10	No drinking	Fluvastatin	Standard	Yes
496	80≃85	Male	Yes	No	No	≥5	5≃10	Heavy drinking	Atorvastatin	Standard	Yes
497	80≃85	Male	No	No	No	≥5	≥10	No drinking	Fluvastatin	Standard	Yes
498	85≃90	Male	No	No	Yes	≥5	≥10	No drinking	Rosuvastatin	Standard	Yes
499	80≃85	Male	No	No	No	3≃5	5≃10	Mild to moderate	Simvastatin	Standard	Yes
500	≥90	Male	No	Yes	No	≥5	5≃10	Mild to moderate	Simvastatin	Standard	Yes
501	80≃85	Male	No	No	No	3≃5	5≃10	No drinking	Atorvastatin	Low	Yes
502	80≃85	Male	Yes	Yes	Yes	≥5	5≃10	Heavy drinking	Fluvastatin	Standard	Yes
503	80≃85	Male	Yes	No	No	3≃5	<5	Mild to moderate	Simvastatin	Low	Yes
504	85≃90	Female	No	No	No	≥5	5≃10	No drinking	Fluvastatin	Standard	Yes
505	80≃85	Female	No	No	No	3≃5	≥10	Mild to moderate	Pravastatin	Standard	Yes
506	80≃85	Male	No	No	No	3≃5	5≃10	Heavy drinking	Fluvastatin	Standard	Yes
507	80≃85	Female	Yes	Yes	No	≥5	5≃10	No drinking	Simvastatin	Standard	Yes
508	≥90	Male	No	No	No	3≃5	<5	No drinking	Fluvastatin	Standard	Yes
509	85≃90	Male	No	No	Yes	≥5	5≃10	Mild to moderate	Pravastatin	Standard	Yes
510	80≃85	Male	No	No	Yes	≥5	<5	Mild to moderate	Pravastatin	Standard	Yes
511	80≃85	Male	No	No	No	≥5	5≃10	No drinking	Atorvastatin	Low	Yes
512	80≃85	Male	Yes	No	No	≥5	<5	Heavy drinking	Atorvastatin	Standard	Yes
513	80≃85	Male	No	No	No	3≃5	5≃10	Mild to moderate	Atorvastatin	Standard	Yes
514	80≃85	Male	No	No	Yes	3≃5	5≃10	Mild to moderate	Fluvastatin	Standard	Yes
515	80≃85	Male	No	No	Yes	≥5	5≃10	Mild to moderate	Pravastatin	Standard	Yes
